# Interaction of Influenza A Viruses with Oviduct Explants of Different Avian Species

**DOI:** 10.3389/fmicb.2017.01338

**Published:** 2017-07-20

**Authors:** Hicham Sid, Sandra Hartmann, Christine Winter, Silke Rautenschlein

**Affiliations:** ^1^Clinic for Poultry, University of Veterinary Medicine Hannover Hannover, Germany; ^2^Institute of Virology, University of Veterinary Medicine Hannover Hannover, Germany

**Keywords:** influenza A virus, poultry, oviduct, antiviral, interferon, H9N2, pH1N1

## Abstract

Infection of poultry with low pathogenic avian influenza viruses (LPAIV) is often associated with mild respiratory symptoms but may also lead to loss in egg production in laying birds. *In vivo* susceptibility of the reproductive tract for LPAIV infection was reported for turkeys and chickens, but virus-interaction with epithelial cells of the oviduct and possible stimulation of the local antiviral immune responses have not been characterized. In this study, we wanted to investigate the suitability of magnum organ cultures (MOC) as an *in vitro* model to study virus-host interactions. We compared the susceptibility of duck (Du), chicken (Ch), and turkey (Tu) MOC for three different influenza A viruses (IAV). Overall, the course of infection and the antiviral immune response varied between strains as well as host cell origin, but MOC gave reproducible results for all investigated parameters within each species. While pandemic (p) H1N1 and H9N2 efficiently replicated in MOC-Ch and MOC-Tu, MOC-Du were significantly less susceptible to infection as indicated by a reduced replication level for both viruses (*p* < 0.05). Overall, virus replication levels did not correlate with interferonα (IFNα) mRNA-expression levels in neither species. H9N2-infection led to a significant upregulation of interferonλ (IFNλ) mRNA expression in MOC of all species compared to the non-infected controls (*p* < 0.05), while a correlation with replication levels was only seen for MOC-Tu. pH1N1-infection induced only significant upregulation of IFNλ mRNA expression in MOC-Tu at 48 hours post infection (*p* < 0.05), but the expression pattern did not correlate with replication levels. Our results show that MOC are a suitable model to study IAV-interaction with the mucosal surface of the avian reproductive tract. The data suggest that the reproductive tract may play a role in the pathobiology of IAV in poultry.

## Introduction

Influenza A viruses (IAV) have been isolated from a wide range of bird species including ducks, turkeys, and chickens but also from mammals (Yoon et al., 2014). Depending on their pathogenicity in specific pathogen free (SPF) chickens, avian influenza viruses (AIV) are divided into highly pathogenic AIV (HPAIV) and low pathogenic AIV (LPAIV). HPAIV are associated with systemic infections and high mortality rates in poultry. Their virulence is influenced by the multibasic cleavage site of the hemagglutinin (HA) which is cleaved by ubiquitous endoproteases including furin and the protein convertase 5/6 allowing systemic spread of the infection (Horimoto et al., 1994; Feldmann et al., 2000). HPAIV have been isolated from different organs including breast muscle, feather follicle, liver, blood, egg content and the oviduct of chickens and Japanese quail (Promkuntod et al., 2006; Beato and Capua, 2011; Silva et al., 2013). LPAIV are of low virulence for most avian species (Alexander, 2000). The HA of most LPAIV has a monobasic cleavage site that needs to be activated by trypsin-like proteases which are believed to be present only in a restricted number of tissues such as the respiratory and intestinal tract (Klenk et al., 1975; Böttcher-Friebertshäuser et al., 2013). It has been shown that *in vitro* infection of various tissue cultures with LPAIV requires exogenous trypsin supplementation for proteolytic activation (Klenk and Rott, 1988; Klenk and Garten, 1994). The infection with IAV is highly dependent on the availability of sialic acids. AIV bind preferentially to α2,3 linked sialic acids while human IAV bind preferentially to α2,6 linked sialic acids. Both linked sialic acids were previously described in different avian species and showed variable expression patterns between species (Kimble et al., 2010; Pillai and Lee, 2010; Mork et al., 2014).

Different IAV-subtypes play an economically important role in poultry species, especially in turkey, being one of the most susceptible poultry species even for LPAIV (Alexander, 2003; Tumpey et al., 2004; Evans et al., 2015). An infection with pandemic H1N1 (pH1N1) was reported in turkey breeders in Chile where birds showed a decline in egg production and shell quality (Mathieu et al., 2010). A different study reported limited susceptibility of turkeys to pH1N1 infection (Kalthoff et al., 2010). Intrauterine infection of turkey hens with pH1N1 was shown to be possible by insemination, which revealed the importance of this route of exposure in turkey production (Pantin-Jackwood et al., 2010). Breeder turkeys infected with H3N2 layed virus-contaminated eggs, which raised concerns regarding AIV dissemination in hatcheries (Pillai et al., 2010). *In vivo* infection of laying hens with H9N2 induced mild hemorrhages in the digestive and respiratory tracts associated with degeneration of epithelial cells and apoptosis in the reproductive tract (Pantin-Jackwood et al., 2012; Wang et al., 2015). In addition, immune-related genes including interleukin-2 (IL-2) and interferonβ (IFNβ) were upregulated (Wang et al., 2015). Similar lesions in the reproductive tract of laying hens were caused by H6N2 in California (Kinde et al., 2003). Meanwhile, H6N2 outbreaks in South African chicken farms were caused by a reassortment between H6N8 and H9N2 (Abolnik et al., 2007). Therefore, we selected three IAV-subtypes of economic importance (H1, H9, and H6) especially for the chicken and turkey production.

The avian reproductive tract can be divided into four different functional parts, the infundibulum, the magnum, the isthmus, and the uterus. Immature chicken oviduct explants were reported to be a successful *in vitro* infection model to study the interaction of infectious bronchitis virus (IBV) with host cells (Mork et al., 2014). Authors concluded that the IBV QX strain replicates efficiently in all oviduct parts. *In vivo* infection studies in chickens with H9N2 indicated that all oviduct sections were susceptible to infection with H9N2 with magnum cells being the most susceptible (Wang et al., 2015). No comparable study was performed in turkeys or Pekin ducks, which are known to show different susceptibility for AIV compared to chickens. While IAV-infection of chickens and turkeys is known to cause a decrease in egg production, no information is available about the interaction of the virus with the reproductive tract of less susceptible species including Pekin ducks. The investigation of IAV-interaction with the reproductive tract of different bird species may provide new insights into the role of the reproductive tract in the pathobiology and epidemiology of IAV. Due to the fact that is difficult to perform such comparative studies under *in vivo* conditions, it would be desirable to establish an *in vitro* model.

The goal of this study was to understand more about the impact of species and IAV strain/subtype variability on host-pathogen interaction at the mucosal surface of the reproductive tract using an *in vitro* model. We used oviduct explants to compare the susceptibility of chicken, turkey, and Pekin duck reproductive tract for infection with three selected viruses, which were speculated to infect at least the turkey reproductive tract: H9N2, pH1N1, and H6N8. A total of three experiments was conducted with respective repeats to compare species and viruses for either replication level and lesions development (Experiments 1 and 3) or interferon expression pattern (Experiment 2, **Table 1**).

**Table 1:** Experimental design.

	Experiment 1 (Exp. 1)	Experiment 2 (Exp. 2)	Experiment 3 (Exp. 3)
Viruses	pH1N1 and H9N2	pH1N1 and H9N2	H6N8
MOC of different species	MOC-Du, MOC-Ch, and MOC-Tu	MOC-Du^∗^, MOC-Ch, and MOC-Tu	MOC-Du and MOC-Tu
Methods	- Immunofluorescence co-staining for detection of IAV-antigen and β-tubulin	- qRT-PCR quantification of viral genome and IFNα and λ mRNA expression	- Immunofluorescence co-staining for detection of IAV-antigen and β-tubulin- Quantification of newly produced viral particles with FFU
			- Quantification of newly produced viral particles with FFU
	- Histology		
	- Quantification of newly produced viral particles with FFU		

*^∗^pH1N1-infected magnum organ cultures (MOC)-Du were not processed for qRT-PCR since no viral antigen was detected with FFU or immunofluorescence staining. FFU, focus forming assay*.

## Materials and Methods

### Oviduct Explants

The oviduct was aseptically isolated from 35-days-old commercial female Pekin ducks (*Anas platyrhynchos domesticus*, Duck-Tec, Belzig, Germany), 12- to 15-week-old layer type SPF chickens (*Gallus gallus domesticus*, VALO BioMedia GmbH, Osterholz-Scharmbeck, Germany) and 12-weeks-old commercial female turkeys (*Meleagris gallopavo* Linnaeus f. domestica, Moorgut Kartzfehn, Bösel, Germany). At least five different birds from the same species were used per experiment. Oviduct explants (15–20/animal) were prepared. Subsequently, rings were randomly selected and transferred into 24-well plates (1 explant/well). Pekin ducks and turkeys were tested negative for antibodies against IAV. All animal experiments were conducted in accordance to the Animal Welfare Regulations of Lower Saxony. In agreement with the German regulations, authorities were notified in advance if animals were killed specifically for tissue collection (“Notification of sacrificing animals for a scientific purpose” from the 11.11.2015, 26.02.2014) or tissues were collected from animals which had been sacrificed for other purposes such as during diagnostic procedures in the diagnostic facilities of the University. Birds were sacrificed according to animal welfare regulations. Oviduct explants were placed immediately in DMEM/Ham’s F-12 medium (Biochrom AG, Berlin, Germany) supplemented with 5% fetal bovine serum (FBS, Biochrom), 2% chicken serum (Sigma–Aldrich, Steinheim, Germany), Penicillin/Streptomycin (P/S; 100 U/ml, 100 mg/ml) (Biochrom), 2.5 ug/ml Amphotericin B (Biochrom) and 1% of non-essential amino acids (Biochrom). The magnum represents the largest part of the oviduct (Rahman, 2014) and was previously reported in chickens to express both α2,3 as well as α2,6 linked sialic acids (Mork et al., 2014), and therefore were speculated that it would be susceptible to avian as well as more human-adapted IAV. The magnum part was manually cut into small rings of 1–2 mm as previously described (Mork et al., 2014). Each magnum explant was subsequently placed in 500 μl medium and infected individually. One hour after infection, medium was replaced and magnum organ cultures (MOC) were incubated with 5% CO_2_ at 37°C. No external trypsin supplementation was used throughout the experiment and media composition was the same between all experimental steps.

### Viruses and Titration

A/chicken/Saudi Arabia/CP7/1998 (H9N2), a field isolate from a meat-type chicken, was propagated in embryonated chicken eggs and titrated in Madin-Darby canine kidney (MDCK) cells as previously described (Balish et al., 2013). The virus had been kindly provided by Hans-Christian Philipp from Lohmann Tierzucht (Cuxhaven, Germany). Pandemic A/Giessen/06/09 (pH1N1), a human isolate, was propagated and titrated in MDCK cells (Balish et al., 2013). A/turkey/Canada/1963 (H6N8) was kindly provided by Klaus Peter Behr from AniCon Labor (Hoeltinghausen, Germany), propagated in embryonated chicken eggs and titrated in MDCK cells. Titration of newly produced viral particles was conducted according to a modified protocol described previously (Baer and Kehn-Hall, 2014). Briefly, MDCK cells were plated in 96-well plates and incubated for 24 h with 5 % CO_2_ at 37°C. Prior to infection, cells were visually evaluated. When 90% confluency was reached, cells were inoculated with 10-fold dilutions of different IAV-subtypes. After 1 h of incubation, the viral inocula were removed and cells were covered with overlay medium for 24 h in the case H9N2 and H6N8 and 36 h for pH1N1. The overlay medium contained 2% DMEM (10x) (Biochrom), 2.5% Avicel^®^ (Sigma–Aldrich), 10 mM HEPES (Sigma–Aldrich), 1 mM Sodium Pyruvate (Biochrom), 0.01% DEAE Dextran hydrochloride (Sigma–Aldrich), P/S (100 U/ml/100 μg/ml) (Biochrom), 0.2% BSA (Carl Roth^®^, Karlsruhe, Germany) and 2 ug/ml trypsin (Biochrom). Cells were fixed with 4% paraformaldehyde supplemented with 1% Triton X (Sigma–Aldrich) for 1 h at room temperature followed by a phosphate-buffered saline (PBS)-washing step. They were processed afterward for primary and secondary antibody staining (Mouse monoclonal anti-nucleoprotein antibodies [clone AA5H] at a dilution of 1:1000 [AbD Serotec, Bio-Rad, Kidlington, United Kingdom], and a polyclonal goat-anti-mouse-IgG/HRP [Abcam, United Kingdom] at a dilution of 1:1000, respectively). Foci formation was visualized by counterstaining with 3-Amino 9-ethylcarbazole (AEC, Sigma–Aldrich) and counted according to the formula described elsewhere (Baer and Kehn-Hall, 2014). pH1N1 and MDCK cells had been kindly provided by Stephan Pleschka, Institute of Medical Virology, Justus-Liebig-Universität (Gießen, Germany). Virus stocks were stored at -70°C.

### Experimental Design

A total of three experiments was conducted. Each experiment was repeated between one and four times, depending on the availability of birds, to confirm the results (**Table 1** and **Supplementary Figure S4**). In Experiments 1 and 2, MOC-Du, MOC-Ch, and MOC-Tu were compared for their susceptibility for pH1N1 and H9N2. Virus replication levels and lesion development (Experiment 1) or cytokine (IFN) mRNA-expression pattern (Experiment 2) were evaluated. In Experiment 3, MOC-Tu and MOC-Du, as a highly and a low susceptible poultry species, were chosen to investigate their susceptibility for H6N8 and to determine virus replication levels and lesion development as compared to Experiment 1. For each species, MOC (*n* = 5/time point) were inoculated with 10^4^ FFU (focus forming units) of H9N2, H6N8 or pH1N1/MOC. The infectious dose was chosen based on preliminary H9N2-infection studies performed in MOC-Ch (data not shown). A similar infection dose was previously used for the infection of tracheal organ cultures (TOC) in this working group (Petersen et al., 2012). Virus-free control MOC (*n* = 5/time point) were incubated with medium only. Both inoculated and virus-free control MOC were incubated for 1 h at 37°C before the supernatant was aspirated and replaced by 1 ml virus-free medium. At different times post infection, MOC were collected and processed for different procedures which included antigen- or cytokine detection by the indicated methods.

### Immunofluorescence Staining for Virus-Antigen and β-Tubulin

MOC were mounted on filter papers using tissue freezing medium (Surgipath^®^, Leica Biosystems Richmond, United States). They were snap-frozen in liquid nitrogen and stored at -70°C. Sections of 7 μm were cut with a Leica cryostat (Nußloch, Germany). IAV nucleoprotein was detected with mouse monoclonal antibodies (clone AA5H) at a dilution of 1:1000 (AbD Serotec, Bio-Rad, Kidlington, United Kingdom). Bound primary antibodies were visualized by Cy3-sheep anti-mouse antibodies (Sigma–Aldrich, Steinheim, Germany) or Alexa fluor^®^ 488 goat anti-mouse antibodies (FITC-labeled, Life Technologies, Carlsbad, CA, United States). Nucleic acid staining was achieved with DAPI, 4′,6-diamidino-2-phenylindole (Life Technologies, Carlsbad, CA, United States). For β-tubulin staining, Cy3-labeled monoclonal antibodies directed against β-tubulin (Clone TUB 2.1, Sigma–Aldrich) were used as previously described (Punyadarsaniya et al., 2011). Fluorescence microscopy was performed with a Nikon Eclipse Ti Microscope. No non-specific staining was seen if slides were only incubated either with the first or second antibody (data not shown).

### Detection of α2,3 and α2,6 Linked Sialic Acids

Detection of α2,3 and α2,6 linked sialic acids was performed using lectins and glycobiology reagents (Vector Laboratories, Burlingame, California, United States). Briefly, endogenous biotin, biotin receptors and streptavidin binding sites were blocked using the Streptavidin/Biotin blocking kit for 15 min. This step was followed by blocking non-specific binding sites by incubation with a carbo-free solution for 30 min. α2,3 linked sialic acids were detected with the biotinylated *Maackia amurensis* lectin II (MAL II). α2,6 linked sialic acids were detected with the biotinylated *Sambucus nigra* lectin (SNA). Both lectins were used at a concentration of 15 μg/ml. Peroxidase activity was developed with the AEC Peroxidase Substrate (Sigma–Aldrich, Germany), following the instructions of the manufacturer. The slides were than counterstained with hematoxylin for 30 s. No non-specific staining was seen, if control slides were processed without the respective lectin (data not shown).

### Histology

Three MOC from each virus-inoculated and virus-free control groups were collected at 12, 24, and 48 hpi and fixed in 4% paraformaldehyde and subsequently embedded in paraffin. MOC were cut into 2 μm sections that were stained with hematoxylin and eosin for histological examination following standard procedures. Examination of the sections addressed histo-pathological changes in the epithelial layer, including degeneration, loss of cilia and hyperplasia of the epithelial cells.

### RNA Isolation and qRT-PCR

Total RNA was isolated with peqGOLD TriFast^TM^ following the instruction guide (PEQLAB Biotechnologie GmbH, Erlangen, Germany).

Quantitative reverse transcription-PCR (qRT-PCR) was conducted with the Ambion AgPath-ID One-Step RT-PCR kit (Life Technologies, Carlsbad, CA, United States) according to the manufacturer’s instructions.

Quantification of AIV was done by the assessment of the M gene and normalization to the 28S rRNA housekeeping gene of the same sample (Δ*C_T_*) as previously described (Petersen et al., 2013). IFNα and IFNλ mRNA expression in chicken and turkey MOC were quantified using the primers and probe that were previously described (Sid et al., 2016). Primers and probes for the detection of IFNα and IFNλ in MOC-Du are presented in **Table 2**. The quantification of IFNα and λ mRNA expression in all investigated species was based on the cycle threshold (CT) values which were normalized against the CT values of the 28S rRNA housekeeping gene as previously described (Petersen et al., 2013).

**Table 2:** List of probes and primers for quantitative real-time reverse transcription-PCR in MOC of Pekin duck (MOC-Du).

RNA target	Primer and probe^a^	Sequence^b^ (5′-3′)	Accession no.^c^
IFNα	IFNα F	AGCTTCAGCACCACATCTAC	EF053034
	IFNα R	TTCTGGAGGAAGTGTTGGATG	
	IFNα P	(FAM)-ACCTTCACCTCAGCACCAACAAGT-(TAMRA)	
IFNλ	IFNλ F	CGGAGGTGCTGAAGTTTAAGA	KJ206897
	IFNλ R	GTGTCCACTTCCGATTGAAGA	
	IFNλ P	(FAM)-TGAGAACATCACGTCGAAGGACCC-(TAMRA)	

^a^*F, forward primer; R, reverse primer; P, probe*.

^b^*FAM, 6-carboxyfluorescein; TAMRA, carboxytetramethylrhodamine*.

^c^*Genomic DNA sequence from GenBank*.

### Statistical Analysis

The Shapiro–Wilk Normality Test was used to test for normal distribution of the data. Statistically significant differences between groups were determined with the two samples *T*-test for normally distributed data and with the Wilcoxon rank-sum test if the data were not normally distributed (Statistix 9.0, Tallahassee, FL, United States). For multiple comparisons between MOC infected with different viruses, we used a Tukey honestly significant differences (HSD) test followed by randomized complete block analysis of variance (ANOVA) for normally distributed data. Not normally distributed data were analyzed with a Kruskal–Wallis one-way ANOVA. Differences were considered significant at *p* < 0.05.

## Results

### Distribution of α2,3 and α2,6 Linked Sialic Acids in MOC-Du, MOC-Ch, and MOC-Tu

**FIGURE 1:**
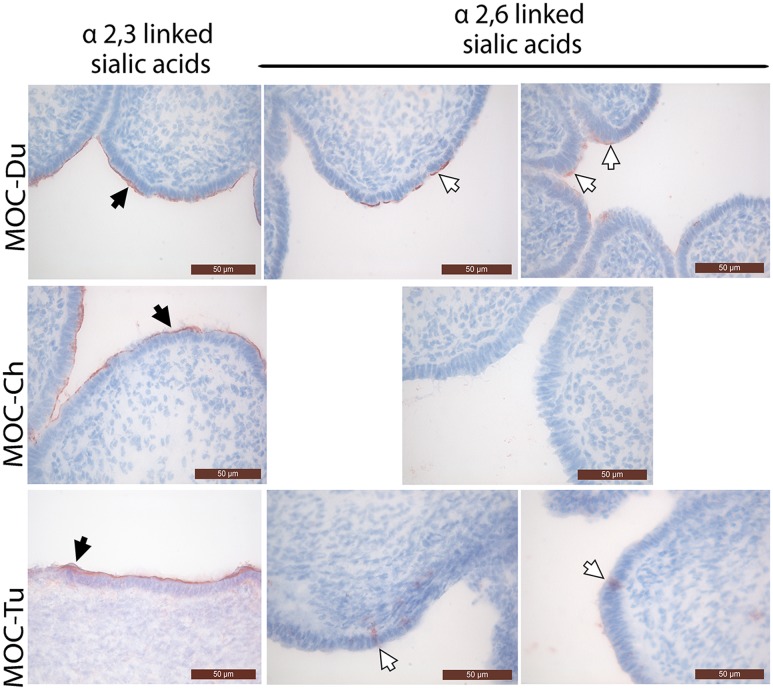
Lectin staining of α2,3 linked sialic acids and α2,6 linked sialic acids in magnum organ cultures (MOC) of Pekin duck (Du), chicken (Ch), and turkey (Tu) (Experiment 1). Virus-free MOC were snap-frozen and processed for lectin staining. White arrows indicate α-2,6 linked sialic acids which were detected with the biotinylated *Sambucus nigra* lectin (SNA). Black arrows indicate α2,3 linked sialic acids which were detected with the biotinylated *Maackia amurensis* lectin II (MAL II). Peroxidase activity was developed with the AEC Peroxidase Substrate. The slides were counterstained with hematoxylin. Data from MOC of two different animals are shown for duck and turkey.

Overall, the mucosal surface of the magnum was dominated by α2,3 linked sialic acids in all investigated species (**Figure 1**). α2,3 linked sialic acids were evenly distributed on the magnum epithelial surface of MOC-Du, MOC-Ch, and MOC-Tu in a comparable manner, although assessment was made only visually. Differences were observed in the expression pattern of α2,6 linked sialic acids between the MOC of the different species. MOC-Du showed the most abundant expression of α2,6 linked sialic acids followed by MOC-Tu and MOC-Ch (**Figure 1**). MOC-Tu showed faint but reproducible staining of only a few cells, which was different to the staining pattern of α2,3 linked sialic acids and was not located on the epithelial surface but more basolateral, the precise location cannot be determined with this staining procedure.

### IAV-Antigen Detection and Lesion Development

**FIGURE 2:**
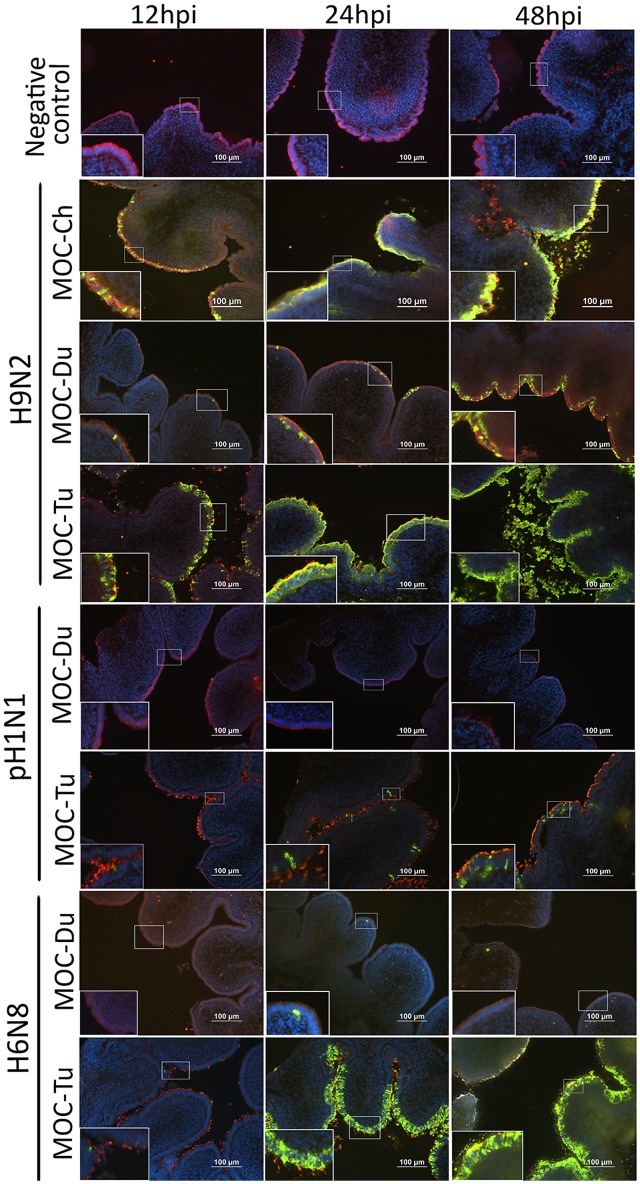
Influenza A viruses (IAV)-antigen detection and β-tubulin staining of MOC of Pekin duck (Du) and turkey (Tu) (Experiments 1 and 3). MOC were infected with H9N2, pH1N1 or H6N8 [10^4^ focus forming assay (FFU)/MOC] and collected at 12, 24, and 48 hours post infection (hpi). MOC sections were stained for IAV-nucleoprotein (FITC, green), β-tubulin (Cy3, red), and cell nuclei (DAPI, blue) and analyzed by fluorescence microscopy. Presented is a merge of three colors: red, green, and blue. IAV nucleoprotein was detected with mouse monoclonal antibodies which were visualized by secondary Alexa fluor^®^ 488 goat anti-mouse antibodies. Negative virus-negative controls consist of non-infected MOC-Ch. A representative picture of each species is shown.

Influenza A viruses-antigen was detected by immunofluorescence staining. Both MOC-Ch and MOC-Tu showed comparable H9N2-staining pattern between 12 and 24 hpi (**Figure 2**). Loss of β-tubulin staining indicated virus-induced epithelial damage in MOC-Tu and MOC-Ch compared to the virus-free controls that exhibited comparable β-tubulin staining throughout all investigated time points. This observation was confirmed by histology, which demonstrated cellular detachment and massive loss of epithelial integrity that was more significant at 48 hpi in MOC-Tu compared to MOC-Ch (**Supplementary Figure S2**). On the other hand, H9N2-infected MOC-Du showed less H9N2 antigen positive epithelial cells at all investigated time points compared to MOC-Ch and MOC-Tu (**Figure 2**). No epithelial cell damage was detected in H9N2-infected MOC-Du compared to the other groups (**Supplementary Figure S2**).

Overall, less pH1N1 antigen positive cells were observed in MOC of all species compared to H9N2-infected MOC. No pH1N1 antigen positive cells were detected in MOC-Du at any investigated time point (**Figure 2**). pH1N1-infected MOC-Ch showed less AIV-antigen positive cells compared to MOC-Tu (**Figure 2** and **Supplementary Figure S1**). pH1N1-antigen was already detected in MOC-Tu at 24 hpi while first pH1N1 positive cells appeared in MOC-Ch at 48 hpi (**Supplementary Figure S1**). pH1N1-infection of MOC-Ch and MOC-Du did not lead to any apparent microscopical epithelial lesions at the investigated time points (data not shown).

Antigen detection pattern of H6N8 in MOC-Tu was comparable to H9N2 while only few H6N8 positive cells were found in MOC-Du (**Figure 2**). Lesion development was only observed in H6N8-inoculated MOC-Tu but not in H6N8-inoculated MOC-Du, which was indicated by inconsistent β-tubulin staining at 48 hpi (**Figure 2**).

### Virus Replication

**FIGURE 3:**
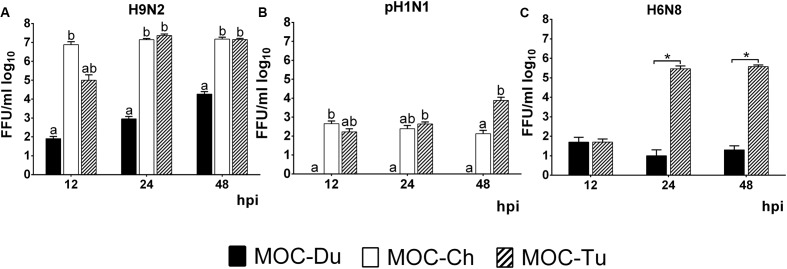
Quantification of H9N2 **(A)**, pH1N1 **(B)**, and H6N8 **(C)** in MOC of Pekin duck (Du), chicken (Ch), and turkey (Tu) by FFU (Experiments 1 and 3). MOC were infected with H9N2 **(A)**, pH1N1 **(B)** or H6N8 **(C)** (infectious dose of 10^4^FFU/explant). Supernatants were collected at 12, 24, and 48 hpi and were subjected to viral titration with the FFU. Results are presented in FFU/ml log_10_. Virus-free controls were negative at all investigated time points (*n* = 5 MOC/group/time point). Different letters indicate differences between groups tested at the same time point post infection *p* < 0.05, ANOVA, Tukey honestly significant differences (HSD). Error bars indicate standard deviation (SD). (^∗^) indicates statistical significance differences between MOC-Tu and MOC-Ch *p* < 0.05, Wilcoxon Rank-Sum Test. A representative repeat of one experiment is shown.

Quantification of infectious virus particles was done by FFU. In the case of H9N2, we observed a significantly lower virus replication level in MOC-Du compared to MOC-Ch and MOC-Tu which confirmed antigen detection data (*p* < 0.05) (**Figure 3**).

In contrast to MOC-Ch and MOC-Tu, MOC-Du were not susceptible to infection with pH1N1 (**Figure 3**). A significant difference in virus replication levels was observed at 48 hpi between MOC-Tu and MOC-Ch (**Figure 3B**).

Quantification of H6N8 by FFU showed a significantly higher viral replication level in MOC-Tu compared to MOC-Du at 24 and 48 hpi (*p* < 0.05) (**Figure 3C**).

In addition, in experiment 2, viral genome copy numbers were quantified by qRT-PCR. Significantly higher numbers of H9N2-genome copies were detected in MOC-Ch compared to MOC-Tu at 12 and 24 hpi (*p* < 0.05), whereas no significant difference was observed between MOC of both species at 48 hpi. Significantly higher pH1N1 genome copies were detected by qRT-PCR in MOC-Tu compared to MOC-Ch at 24 and 48 hpi (*p* < 0.05) (**Supplementary Figure S3**).

### Antiviral Gene Expression

mRNA gene expression of IFNα and IFNλ was investigated in experiment 2 in MOC of all species. These cytokines were selected as representative of the antiviral innate immune response, being especially associated with virus infections of epithelial cells (Reuter et al., 2014; Sid et al., 2016; Santhakumar et al., 2017). Due to lack of detectable pH1N1-replication in MOC-Du, IFN-mRNA expression was not investigated in pH1N1-inoculated MOC-Du. Overall, MOC-Tu showed the highest upregulation of IFNα and IFNλ at 12, 24, and 48 h post H9N2-infection, and for IFNλ at 48 h post pH1N1-infection compared to the MOC-Ch and MOC-Du (*p* < 0.05).

**FIGURE 4:**
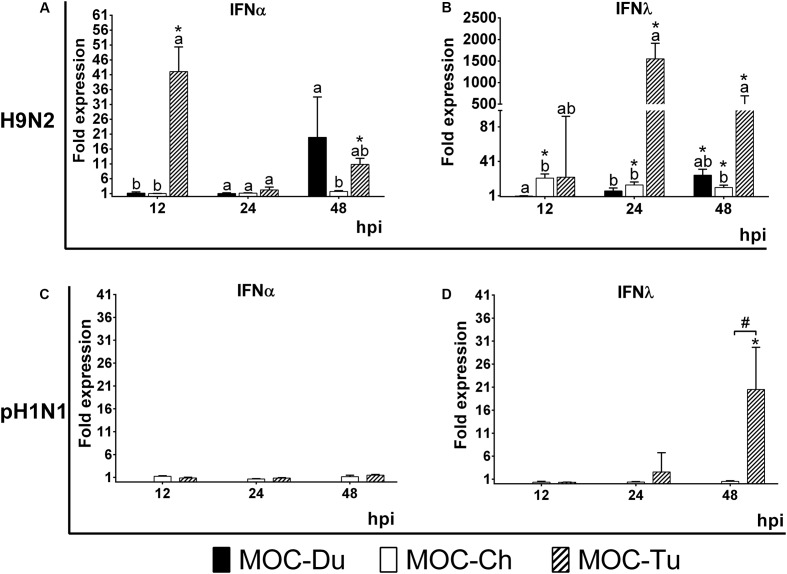
Interferon (IFN) α **(A,C)** and λ **(B,D)** mRNA expression in MOC of Pekin duck (Du), chicken (Ch), and turkey (Tu) after infection with H9N2 **(A,B)** and pH1N1 **(C,D)** (Experiment 2). IFNα and IFNλ mRNA expression of infected MOC are presented in fold-change compared to the respective virus-free controls. MOC were collected at 12, 24 and 48 hpi and processed for RNA isolation and quantification of IFNα and IFNλ mRNA expression by qRT-PCR. pH1N1-infected MOC-Du were not susceptible to infection as demonstrated by FFU and antigen detection by immunofluorescence, therefore no IFN quantification of was conducted. Values were normalized to 28S rRNA expression (Petersen et al., 2013) (*n* = 5 MOC/group/time point). MOC had been infected with pH1N1 and H9N2 with an infectious dose of 10^4^ FFU/explant. Different letters indicate differences between groups tested at the same time point post infection *p* < 0.05, Kruskal–Wallis All-Pairwise Comparisons Test. (^∗^) indicate statistical significance between virus-infected and virus-free controls *p* < 0.05, two sample *T*-test. (#) indicates statistical significance between MOC-Tu and MOC-Ch *p* < 0.05, two sample *T*-test. Error bars indicate SD. A representative repeat of Experiment 2 is shown.

Interferonα mRNA expression was significantly increased in H9N2-infected MOC-Tu by 42 and 10-fold at 12 and 48 hpi, respectively, compared to the virus-free controls (*p* < 0.05) (**Figure 4A**). H9N2-infection also induced significant upregulation of IFNα mRNA expression by 19-fold in MOC-Du at 48 hpi (*p* < 0.05) while no change in IFNα mRNA expression was observed in H9N2-infected MOC-Ch compared with virus-free MOC (*p* > 0.05). pH1N1-infection did not lead to any changes in the IFNα gene expression in investigated MOC compared to the virus-free controls (**Figure 4C**).

H9N2-infected MOC-Du did not show significant upregulation of IFNλ mRNA expression up to 24 hpi. At 48 hpi, the endpoint of the experiment, we detected a 25-fold increase compared to the virus-free controls (*p* < 0.05) (**Figure 4B**). H9N2-infected MOC-Tu showed significant upregulation of IFNλ mRNA expression up to more than 1500-fold at 24 hpi compared to the virus-free controls (*p* < 0.05) (**Figure 4B**). IFNλ mRNA upregulation was also observed at all investigated time points after H9N2-infection of MOC-Ch compared to the virus-free controls (*p* < 0.05).

Infection with pH1N1 only led to significant IFNλ upregulation in MOC-Tu with an up to 21-fold increase at 48 hpi compared to virus-free controls (**Figure 4D**). No significant changes were observed in IFNλ mRNA expression in pH1N1-infected MOC-Ch.

## Discussion

So far, only little was known about the role of the avian reproductive tract in IAV pathogenesis. In order to understand more about the interaction of IAV with the mucosa of the reproductive tract of avian hosts and to identify the impact of species and virus strain/subtype variability on the infection, we used MOC as an *in vitro* model to compare the tissues of the selected poultry species chicken, turkey and duck under the same experimental conditions. The interaction of three selected IAV strains of different subtypes including H9N2, H6N8, and pH1N1 with the epithelial layer of the oviduct of the three avian species was compared by looking at virus replication level, lesion development and the antiviral immune response.

Overall, MOC were collected from immature female birds. Conducting experiments with immature oviduct explants allowed avoiding physiological-related differences between bird species (Mohammadpour, 2007). It has to be recognized that the maturation of the oviduct may have been different between the bird species because of different age at the time of tissue collection. As demonstrated previously, age seems to have no significant or only minor influences on the distribution and staining intensity of α2,3 and α2,6 sialic acids (Pillai and Lee, 2010), which are important receptors of IAV, therefore we consider that the differences in age may be negligible with respect to the objectives of our study. LPAIV-infection of cells *in vitro* usually requires exogenous trypsin in order to ensure proteolytic cleavage of HA0 into HA1 and HA2 (Klenk and Garten, 1994). Our study is the first to report that IAV replicate in the avian reproductive explants without the need for exogenous proteolytic activation. This may suggest the presence of endogenous proteases in the epithelial cells of the reproductive tract being capable of cleaving monobasic cleavage sites (Lim et al., 2012), which has to be investigated in future studies.

We investigated the dynamics of IAV-infection at 12, 24, and 48 hpi by antigen detection with immunofluorescence staining, FFU assay and quantification by real-time RT-PCR. Overall, MOC-Du were shown, with all three methods, to be less susceptible *in vitro* for IAV-infection compared to MOC-Ch and MOC-Tu independent of the investigated influenza subtype. *In vivo* studies previously reported that ducks were less susceptible to LPAIV in comparison to other birds including domestic poultry, jungle crows, and tree sparrows (Hiono et al., 2016); whereas, authors did not investigate the reproductive tract. Possible differences in viral binding and replication of human-adapted virus pH1N1 and avian adapted viruses H9N2 and H6N8, could be the variation in the expression pattern of α2,3 and α2,6 sialic acids in the epithelial layer (Pillai and Lee, 2010). It is believed that human influenza viruses including pH1N1 bind preferably to α2,6 linked sialic acids whereas AIV including H9N2 preferentially bind to α2,3 linked sialic acids (Wan and Perez, 2006). Our results are in agreement with previously published data that indicated higher expression of α2,3 linked sialic acids in all parts of the chicken oviduct compared to α2,6 linked sialic acids which were less expressed particularly in the chicken magnum (Mork et al., 2014; Wang et al., 2015). This difference in receptor abundance may subsequently influence the replication level of different IAV (Suzuki et al., 2000).

The sialic acid detection method that we used varied in comparison to other studies. Immunohistochemical detection in our study showed lower number of α2,6 linked sialic acid-positive cells in MOC-Ch compared to the same structures investigated by Mork et al. (2014) using an immunofluorescence-based method. In addition, other differences including genotype of the birds used in the study could be different which may explain the slight variation between staining pattern.

In contrast to previous studies by Pillai and Lee (2010), MOC-Du showed α2,6 linked sialic acid-positive cells. But interestingly despite α2,6 linked sialic acid-positive cells, no pH1N1-infection was detected, which was different to MOC-Ch that showed pH1N1-infected cells in the absence of α2,6 linked sialic acid-positive cells. This may suggest that other factors may be involved in the control of virus replication in the reproductive tract of the different bird species which should be further investigated. Differences between chickens and ducks were previously described in innate immune response mechanisms including the expression of RIG-I and RNF 135 (Smith et al., 2015).

We observed differences in the localization of α2,3 linked sialic acids and α2,6 linked sialic acids staining between species, which was more luminal in MOC-Ch and MOC-Du, but luminal and lateral in MOC-Tu, respectively. Further investigations are necessary to determine the exact location of α2,6 linked sialic acids in MOC-Tu. Overall this suggests species specific differences, which may subsequently affect the susceptibility for IAV-infection. Similar observations about the more lateral distribution pattern of α2,6 linked sialic acids in MOC-Tu have been made in pigs (Van Poucke et al., 2010; Punyadarsaniya et al., 2011; Trebbien et al., 2011). We may speculate that under field conditions, other pathogens lead to a destruction of the epithelial layer, making basolateral receptors more accessible (Rudd et al., 2016). Under *in vitro* conditions, these structures may be accessible due to the fact that MOC were excised and epithelial monolayer was not fully intact throughout the organ explant. Overall, our three detection methods including antigen staining, viral replication and genome copy numbers reflected the differences in IAV-susceptibility between MOC-Ch, MOC-Du, and MOC-Tu. At 12 hpi, results of the qRT PCR indicated significantly higher H9N2-genome copy numbers in MOC-Ch compared to MOC-Tu and MOC-Du, while no significant difference was observed between MOC-Tu and MOC-Ch at 24 and 48 hpi. This demonstrates that early H9N2-replication may be faster in the chicken oviduct compared to turkey; later on, the lack of target cells in MOC-Ch may lead to comparable FFU and Ct values between species. Our data also indicated less efficient replication of pH1N1 compared to H9N2 and H6N8 in MOC-Ch and MOC-Tu while no replication of pH1N1 was detected in MOC-Du. In contrast to H9N2 and H6N8, pH1N1-inoculation did not lead to major destruction of the magnum epithelium of MOC-Ch and MOC-Tu up to 48 hpi, the last time point of investigation for MOC-Tu and 72 hpi, the last time point of investigation for MOC-Ch. This suggests that virus replication level may correlate with lesion development.

We further quantified the IFN mRNA expression pattern after infection with pH1N1 and H9N2 at 12, 24, and 48 hpi. Overall, there was no clear correlation between H9N2-replication and IFN-expression levels for MOC-Ch and MOC-Du at neither of the investigated time points. pH1N1-replication levels were low in comparison to H9N2 in both species, and no IFN-expression was detected. Interestingly, H9N2 replicated to high levels in MOC-Tu, which also showed high IFNλ expression levels in all investigated time points, indicating a possible correlation. On the other hand, there was no clear correlation between H9N2-replication rates and IFNα mRNA-expression levels, which was also shown in a different study conducted in TOC of turkey with different IAV-subtypes (Petersen et al., 2013). There was a clear upregulation of IFNλ mRNA expression after pH1H1-infection of MOC-Tu at 24 and 48 hpi; although virus levels had also increased, a clear correlation cannot be seen between both parameters. At this point there is no explanation behind the different regulation mechanisms of the IFN-response in MOC. However, differences in cytokine expression pattern in relation to the virus replication levels between MOC of different birds species support the observation that innate immune reactions may vary between bird species (Cornelissen et al., 2012; Zhang et al., 2015)

The upregulation of IFNα mRNA expression in MOC-Tu at 12 hpi was unexpected with respect to the expression levels at the later time points (24 and 48 hpi). This may be due to inflammatory reactions caused by excision of oviduct explants into rings. A similar observation was reported after mechanic excision of TOC (Reemers et al., 2009). Authors stated that TOC preparation led to early upregulation of IL-6, IL-8, and IL-10.

In our study, pH1N1 did not induce any significant upregulation of IFNα mRNA expression in MOC of either species. Infection of primary chicken lung cells with pH1N1 also did not lead to IFNα upregulation expression while infection with H5N9 led to a significant IFNα upregulation (Jiang et al., 2011). We may speculate that AIV, which are more adapted to chicken epithelial cells, may induce a higher IFN-type I response than human-adapted influenza viruses in bird tissue. This may be due to a different receptor spectrum between mammalian and avian species and subsequently a different replication level (Nicholls et al., 2007; Schrauwen and Fouchier, 2014).

mRNA expression of IFNλ in the avian reproductive tract had not been investigated before. Our results demonstrated that the H9N2-infection of MOC of all investigated species led to a significant upregulation of IFNλ mRNA expression compared to the virus-free controls. On the other hand, only pH1N1-infected-MOC-Tu demonstrated a significant upregulation of IFNλ mRNA expression at 48 hpi compared to the virus-free controls (*p* < 0.05) while no change in expression was observed in MOC-Ch. The protective role of IFNλ against AIV in the upper respiratory tract of chickens was previously described (Reuter et al., 2014). In mice, IFNλ was shown to be highly efficient in preventing respiratory and gastrointestinal tract infections with pathogens such as influenza viruses, human metapneumovirus, and severe acute respiratory syndrome (SARS) coronavirus (Mordstein et al., 2010). Although we detected high expression of IFNλ mRNA expression in MOC-Tu, this species was highly sensitive to IAV-infection. Further studies should be conducted to investigate the protein function of IFNλ and its receptor in the avian reproductive tract. In addition, further cytokines being possibly involved in the innate immune response against IAV may be investigated in the future, to obtain a more complete picture about the virus-host interaction at the epithelial surface of the avian reproductive tract.

Overall, our study demonstrated that there is a significant difference in the susceptibility of the reproductive tract for IAV between different avian species, and that the infecting strain/subtype may influence the infection outcome. MOC is a suitable *in vitro* model to investigate the avian reproductive tract for virus-host interactions. It allowed the comparison of MOC derived from different bird species as well as different viruses. MOC from other bird species including wild aquatic birds may also be used in the future to investigate their possible role in IAV shedding via the reproductive tract (Krauss and Webster, 2010; Hénaux and Samuel, 2011). In addition, *in vivo* studies may be needed to confirm the *in vitro* results.

## Ethics Statement

The authorities (Lower Saxony State Office for Customer Protection and Food Safety) were notified when animals were specifically sacrificed for the purpose of organ collection. The sacrificing was conducted in accordance to the Animal Welfare Regulations of Lower Saxony.

## Author Contributions

SR: Planned the study and experiments, evaluated the results, and prepared the manuscript. HS: Planned and conducted the experiments, evaluated the results, and prepared the manuscript. CW: Established methods and evaluated the results. SH: Conducted the experiments and evaluated the results.

## Conflict of Interest Statement

The authors declare that the research was conducted in the absence of any commercial or financial relationships that could be construed as a potential conflict of interest.

## Supplementary Material

FIGURE S1 IAV-antigen detection in MOC of chicken (Ch) (Experiment 1). MOC-Ch were infected with pH1N1 (10^4^ FFU/MOC) and collected at 12, 24, 48, and 72 hours post infection (hpi). MOC sections were stained for pH1N1 (Cy3, red) and cell nuclei (DAPI, blue). MOC-Ch were analyzed by fluorescence microscopy. IAV nucleoprotein was detected with mouse monoclonal antibodies which were visualized by secondary Cy3-labeled sheep anti-mouse antibodies. Presented is a representative picture from each time point.

FIGURE S2 Lesion development in MOC of Pekin duck (Du), chicken (Ch), and turkey (Tu) (Experiment 1). MOC-Du, MOC-Ch, and MOC-Tu were infected with H9N2 (infectious dose of 10^4^ FFU/MOC) and collected at 12, 24, and 48 hpi and subsequently processed for histology.

FIGURE S3 Quantification of H9N2 **(A)** and pH1N1 **(B)** in MOC of Pekin duck (Du), chicken (Ch), and turkey (Tu) by qRT-PCR (Experiment 2). MOC were infected with H9N2 or pH1N1 (infectious dose of 10^4^FFU/explant). They were collected at 12, 24, and 48 hpi and processed for RNA isolation and quantification of the M gene by qRT-PCR. Threshold (Ct) values are presented, which were normalized against the CT values of the 28S rRNA housekeeping gene of the same sample (Δ*C_T_*) (Petersen et al., 2013). Different letters indicate differences between groups tested at the same time point post infection *p* < 0.05, Kruskal–Wallis All-Pairwise Comparisons Test. pH1N1-infected MOC-Du were not susceptible to productive infection as demonstrated by FFU and antigen detection by immunofluorescence staining, therefore no qRT-PCR was performed **(B)** (^∗^) indicates statistical significance differences between virus-infected and virus-free controls *p* < 0.05, Wilcoxon Rank-Sum Test (*n* = 5 MOC/group/time point). Error bars indicate standard deviation (SD). A representative repeat of Experiment 2 is shown.

FIGURE S4 Repeat experiment of MOC-Tu infected with H9N2 and pH1N1. MOC were infected with H9N2 and pH1N1 (infectious dose of 10^4^FFU/explant). Supernatants were collected at 12, 24, and 48 hpi and were subjected to viral titration with the focus forming assay (FFU) **(A)**. IFNα and IFNλ mRNA expression of infected MOC-Tu are presented in fold-change compared to the respective virus-free controls following H9N2 and pH1N1 infections (**B,C**, respectively). Wilcoxon Rank-Sum Test (*n* = 5 MOC/group/time point). Error bars indicate standard deviation (SD). (^∗^) indicates statistical significance differences between virus-infected and virus-free controls *p* < 0.05.
