# Supplementary figures and images for: Interaction of Influenza A Viruses with Oviduct Explants of Different Avian Species
Source: Front Microbiol. 2017 Jul 20;8:1338. doi: 10.3389/fmicb.2017.01338 (PMC5518544; doi:10.3389/fmicb.2017.01338)

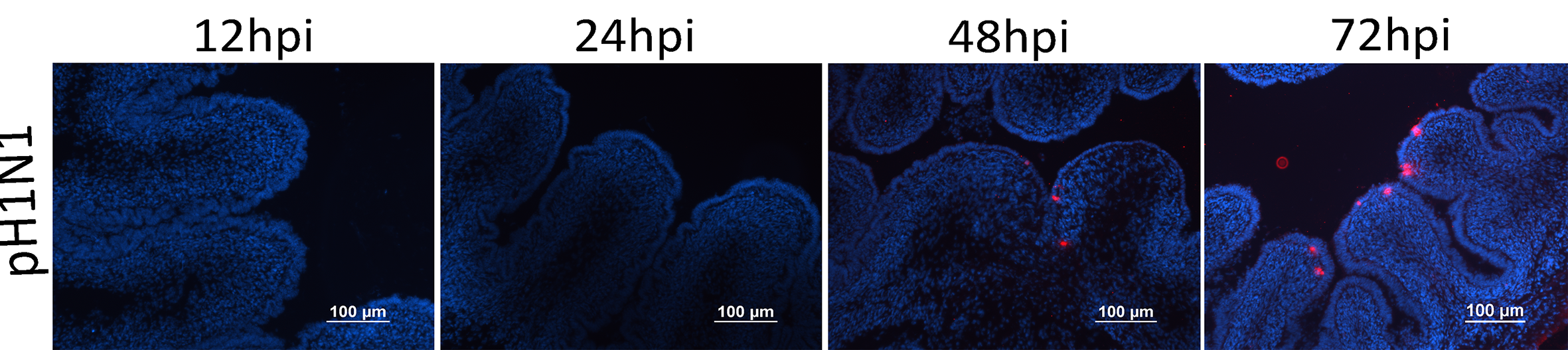

Supplement: FIGURE S1 — IAV-antigen detection in MOC of chicken (Ch) (Experiment 1). MOC-Ch were infected with pH1N1 (104 FFU/MOC) and collected at 12, 24, 48, and 72 hours post infection (hpi). MOC sections were stained for pH1N1 (Cy3, red) and cell nuclei (DAPI, blue). MOC-Ch were analyzed by fluorescence microscopy. IAV nucleoprotein was detected with mouse monoclonal antibodies which were visualized by secondary Cy3-labeled sheep anti-mouse antibodies. Presented is a representative picture from each time point. [file Image_1.TIF]

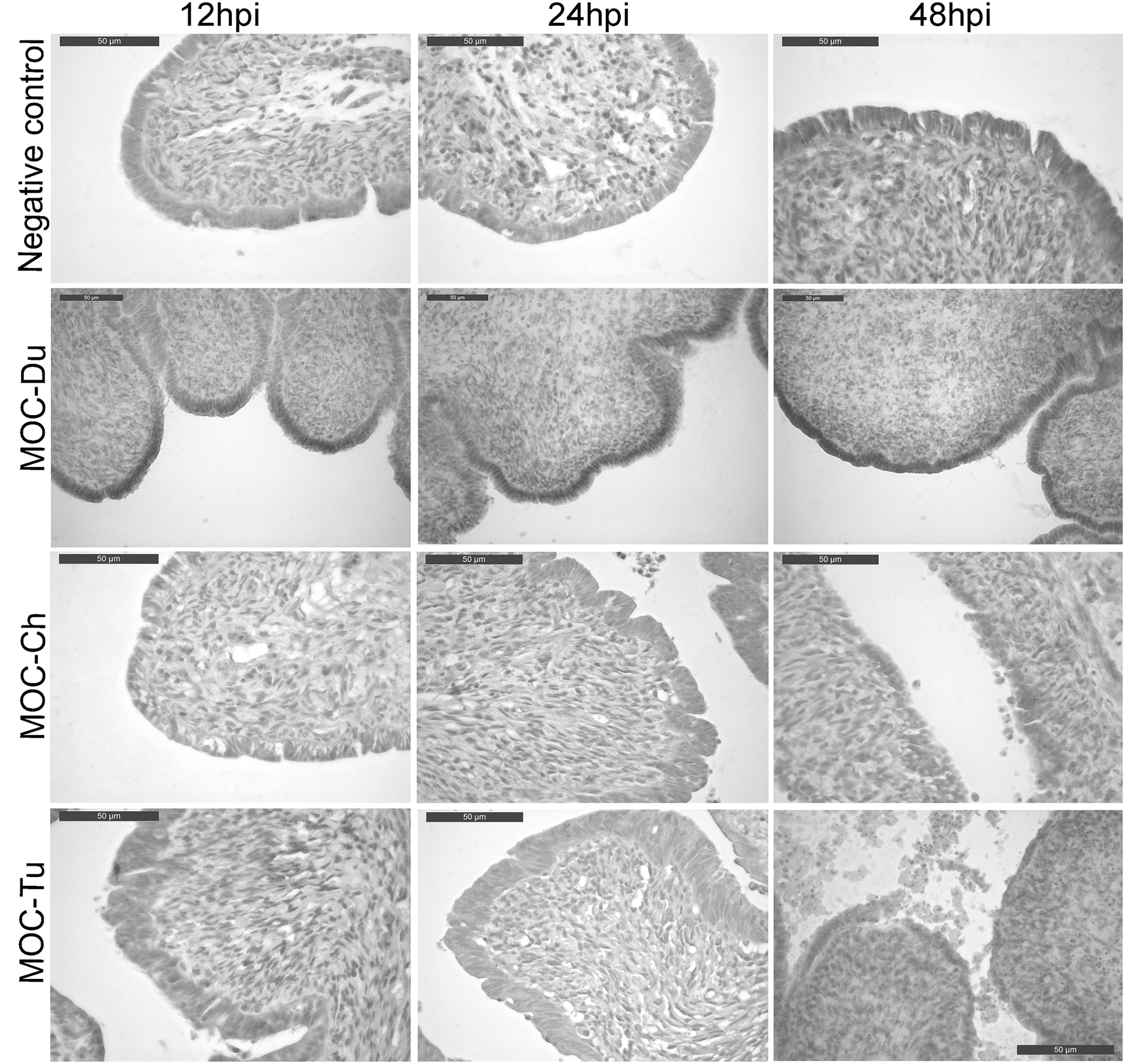

Supplement: FIGURE S2 — Lesion development in MOC of Pekin duck (Du), chicken (Ch), and turkey (Tu) (Experiment 1). MOC-Du, MOC-Ch, and MOC-Tu were infected with H9N2 (infectious dose of 104 FFU/MOC) and collected at 12, 24, and 48 hpi and subsequently processed for histology. [file Image_2.TIF]

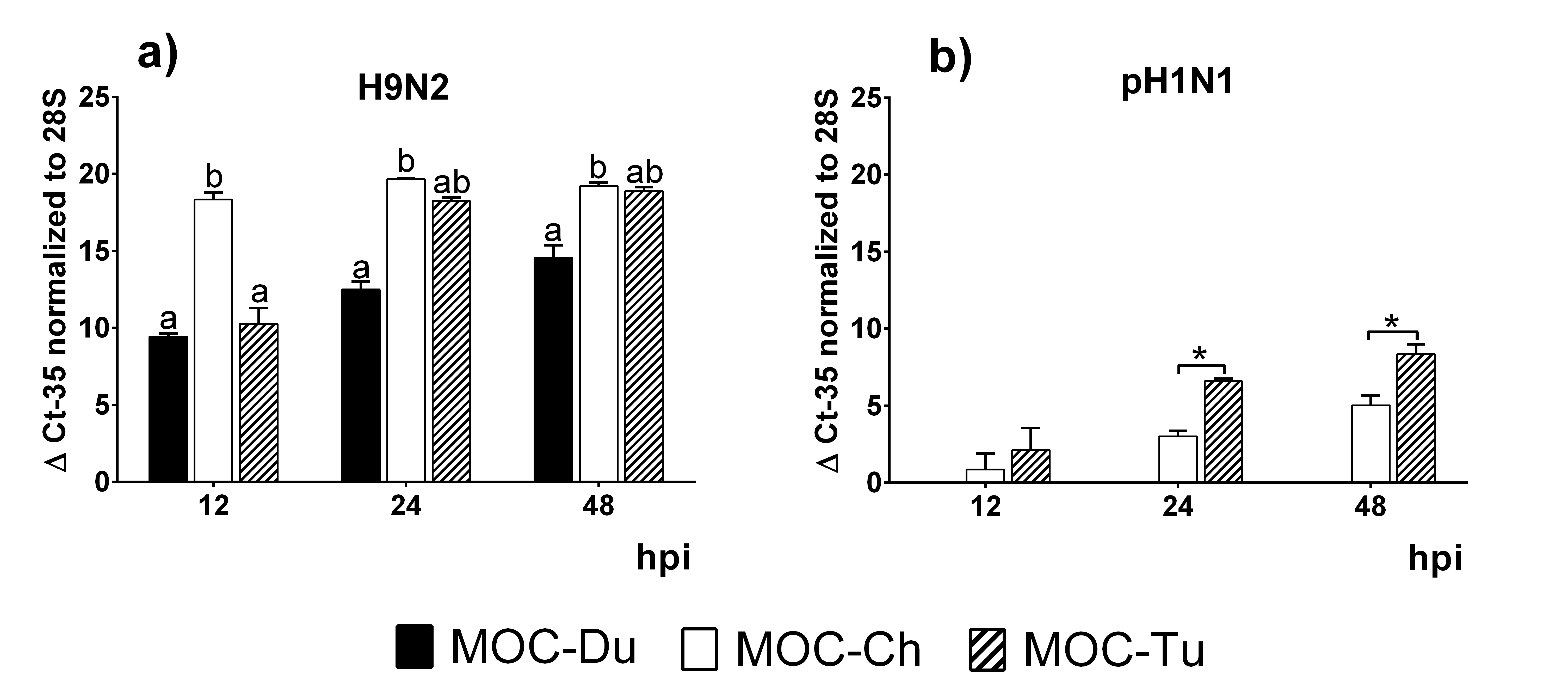

Supplement: FIGURE S3 — Quantification of H9N2 (A) and pH1N1 (B) in MOC of Pekin duck (Du), chicken (Ch), and turkey (Tu) by qRT-PCR (Experiment 2). MOC were infected with H9N2 or pH1N1 (infectious dose of 104FFU/explant). They were collected at 12, 24, and 48 hpi and processed for RNA isolation and quantification of the M gene by qRT-PCR. Threshold (Ct) values are presented, which were normalized against the CT values of the 28S rRNA housekeeping gene of the same sample (ΔCT) (Petersen et al., 2013). Different letters indicate differences between groups tested at the same time point post infection p < 0.05, Kruskal–Wallis All-Pairwise Comparisons Test. pH1N1-infected MOC-Du were not susceptible to productive infection as demonstrated by FFU and antigen detection by immunofluorescence staining, therefore no qRT-PCR was performed (B) (∗) indicates statistical significance differences between virus-infected and virus-free controls p < 0.05, Wilcoxon Rank-Sum Test (n = 5 MOC/group/time point). Error bars indicate standard deviation (SD). A representative repeat of Experiment 2 is shown. [file Image_3.TIF]

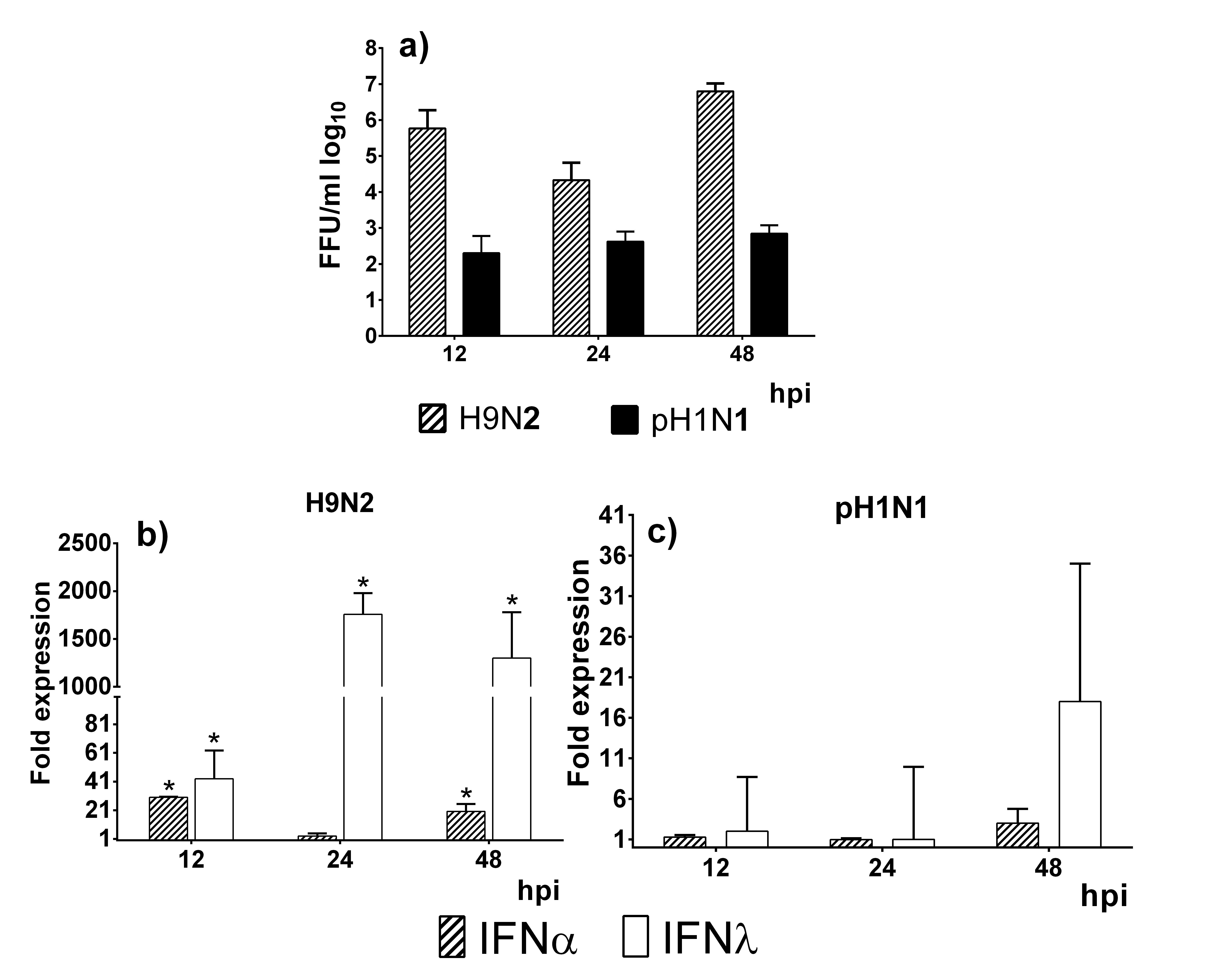

Supplement: FIGURE S4 — Repeat experiment of MOC-Tu infected with H9N2 and pH1N1. MOC were infected with H9N2 and pH1N1 (infectious dose of 104FFU/explant). Supernatants were collected at 12, 24, and 48 hpi and were subjected to viral titration with the focus forming assay (FFU) (A). IFNα and IFNλ mRNA expression of infected MOC-Tu are presented in fold-change compared to the respective virus-free controls following H9N2 and pH1N1 infections (B,C, respectively). Wilcoxon Rank-Sum Test (n = 5 MOC/group/time point). Error bars indicate standard deviation (SD). (∗) indicates statistical significance differences between virus-infected and virus-free controls p < 0.05. [file Image_4.TIF]
